# Mother–Infant Brain-to-Brain Synchrony Patterns Reflect Caregiving Profiles

**DOI:** 10.3390/biology12020284

**Published:** 2023-02-10

**Authors:** Yaara Endevelt-Shapira, Ruth Feldman

**Affiliations:** 1 Center for Developmental Social Neuroscience, Reichman University, Herzliya 4610101, Israel; 2 Child Study Center, Yale University, New Haven, CT 06520, USA

**Keywords:** inter-brain synchrony, maternal sensitivity, intrusiveness, social brain, social neuroscience, hyperscanning

## Abstract

**Simple Summary:**

Sensitive caregiving implies the mother’s moment-by-moment adaptation to the infant’s states and signals, and such online coordination supports the child’s social and neurobiological development. In contrast, intrusive mothering is characterized by overstimulation and social interactions guided by the maternal agenda rather than the infant’s interactive cues. These two maternal behavioral styles have been extensively studied and repeatedly shown to predict positive and negative social-emotional outcomes, respectively. Here we show that these two styles, sensitivity and intrusiveness, are differentially related to mechanisms of mother–infant brain-to-brain synchrony; while sensitivity is linked with higher mother–infant neural synchrony, intrusiveness is associated with diminished inter-brain coordination. We believe that the enhancement or limitation on coordinated interactive inputs to the infant’s social brain during its maturational period may be one mechanism by which maternal sensitivity and intrusiveness exert their differential long-term effects on children’s brains and behaviors.

**Abstract:**

Biobehavioral synchrony, the coordination of physiological and behavioral signals between mother and infant during social contact, tunes the child’s brain to the social world. Probing this mechanism from a two-brain perspective, we examine the associations between patterns of mother–infant inter-brain synchrony and the two well-studied maternal behavioral orientations—sensitivity and intrusiveness—which have repeatedly been shown to predict positive and negative socio-emotional outcomes, respectively. Using dual-electroencephalogram (EEG) recordings, we measure inter-brain connectivity between 60 mothers and their 5- to 12-month-old infants during face-to-face interaction. Thirty inter-brain connections show significantly higher correlations during the real mother–infant face-to-face interaction compared to surrogate data. Brain–behavior correlations indicate that higher maternal sensitivity linked with greater mother–infant neural synchrony, whereas higher maternal intrusiveness is associated with lower inter-brain coordination. Post hoc analysis reveals that the mother-right-frontal–infant-left-temporal connection is particularly sensitive to the mother’s sensitive style, while the mother-left-frontal–infant-right-temporal connection indexes the intrusive style. Our results support the perspective that inter-brain synchrony is a mechanism by which mature brains externally regulate immature brains to social living and suggest that one pathway by which sensitivity and intrusiveness exert their long-term effect may relate to the provision of coordinated inputs to the social brain during its sensitive period of maturation.

## 1. Introduction

Since Mary Ainsworth first described the mother’s sensitive style as the central vehicle for the development of infant attachment security [1], thousands of studies have pinpointed maternal sensitivity as a key contributor to children’s social-emotional growth. Extant research in healthy and high-risk populations and across diverse cultures, as well as several meta-analyses [2,3,4], provided a solid proof to the claim that the experience of sensitive caregiving across the first year of life is critical for the development of child socialization, emotional skills, and well-being. Longitudinal studies have further shown that maternal sensitivity is not only a direct predictor of attachment security but also shapes the child’s global social competencies [5,6], emotion regulation [7,8], peer relationships, school achievements [6,9], and behavioral adaptation [10,11] across the toddler and preschool years and up until adolescence [5,6]. Follow-up studies in the field of attachment during the 1980s [12,13] and 1990s [14] further probed the relational precursors of attachment security and insecurity and addressed “maternal intrusiveness” as an antecedent of the infant’s insecure-anxious attachment [12] and as a predictor of behavior problems, dysregulation, social maladjustment [15], and disrupted language development [16,17,18]. Across studies, maternal sensitivity was defined as the mother’s warmth and acceptance, appropriate stimulation, acknowledgement of the infant’s interactive cues, and co-regulation of the social exchange, whereas intrusiveness was characterized by maternal control, over-stimulation, and disregard of the infant’s overall state and nonverbal signals. 

In searching for the mechanisms by which maternal sensitivity and intrusiveness exert their long-term effect, one perspective considers their individual stability across lengthy developmental epochs [19]. Both maternal sensitivity and intrusiveness were found to be individually stable over time [20], including studies that measured their consistency from infancy to adolescence [21] and young adulthood [22], suggesting that it is the repeated experience of sensitive versus intrusive mothering, not momentary variations, that renders their pervasive effect. Another possible explanation relates to their differential impact on the infant’s neurohormonal maturation, particularly on biological systems involved in stress management and affiliation. Sensitive parenting has been repeatedly associated with higher levels of child oxytocin [23,24,25], whereas intrusive parenting has been linked with elevated HPA-axis activation and greater stress response in infants and children [26,27]. In the current study, we suggest that sensitivity and intrusiveness exert a differential effect on the infant’s social brain during its sensitive period of maturation and utilize, for the first time, a two-brain approach to test this hypothesis. 

Overall, the neural mechanisms by which maternal sensitivity and intrusiveness make their lasting impact on the child’s brain are not fully clear. Studies have shown that maternal sensitivity and intrusiveness longitudinally impact the child’s brain, particularly the brain basis of social functions. For instance, intrusive mothering at 9 months was found to predict a disrupted neural empathic response to others’ pain in adolescence, as measured by magnetoencephalography (MEG) [28]. In another MEG study, sensitive mothering longitudinally predicted the brain’s response to attachment cues [29], as well as the adolescent’s neural empathic response to others’ emotional distress [30]. The mother’s sensitive and synchronous style, repeatedly measured from infancy to adulthood, was further found to predict young adults’ neural sensitivity to others’ distinct emotions (i.e., empathic accuracy), as measured by fMRI [22]. In addition to the brain basis of social functions, intrusive mothering in infancy was longitudinally associated with disruptions in default mode network (DMN) connectivity in adolescence in two longitudinal cohorts [31,32], suggesting that sensitivity and intrusiveness not only shape the brain’s social functions but also impact the consolidation of the brain’s fundamental architecture. 

In the current study, we approach this question from a two-brain perspective. We examine whether maternal sensitivity and intrusiveness are associated with distinct patterns of inter-brain connectivity between mother and infant that may be more or less beneficial for the infant’s brain maturation. During the second six months of life, the “social brain” undergoes rapid maturation in terms of structural [33] and functional modifications [34,35], the development of networks that enable attention and emotion regulation, and the consolidation of the default-mode [35] and frontoparietal theta networks [36], and we therefore targeted our study at that period.

The study was guided by the biobehavioral synchrony conceptual frame, which suggests that maternal behavior characterized by ongoing coordination with the infant’s nonverbal signals and couched within a well-adapted, fluent, and reciprocal dialogue (i.e., maternal sensitivity) provides the template for the coordination of the physiological processes between mother and child, including heart rhythms, hormonal release, or inter-brain synchrony [37,38,39]. Such biobehavioral linkage is a critical social input the infant must experience during the first months of life, prior to the onset of language and the first major reorganization of the prefrontal cortex [37,40]. Here, we examined the hypothesis that the mother’s sensitive style is associated with greater inter-brain synchrony, whereas the intrusive style is linked with diminished neural synchrony, and that such differences in coordinated neural input may exert a lasting effect on the infant’s brain and behavior.

Consistent with prior mother–child hyperscanning studies, we were particularly interested in neural connectivity between the mother’s frontal region and the infant’s temporal region. Previous research has shown that caregiver–child inter-brain synchrony typically engages frontal, parietal, and temporal regions [41,42,43,44,45], and the connectivity between frontal and temporal regions has been repeatedly found in cross-brain studies between mothers and young children. For instance, mothers and their 4–6-year-old children exhibited neural synchrony in temporo-parietal and prefrontal areas during a free verbal conversation [41]. Similarly, a dual-fNIRS study revealed that during a problem-solving task, mothers and their preschool-aged children showed enhanced brain-to-brain synchrony in the cooperative condition relative to a condition when mothers and their children solved the puzzle individually in bilateral prefrontal and temporo-parietal regions. Furthermore, when mothers and children acted more reciprocally during the cooperation task, higher neural synchrony in these regions was detected and maternal self-reported stress correlated with diminished neural synchrony [42]. Notably, maternal stress has been repeatedly linked with the intrusive maternal style [46]. An ecological hyperscanning study of infant–mother and infant–stranger interactions showed inter-brain connectivity with the infant’s right temporal region during both mother–infant and stranger–infant paradigms [43]. Finally, a recent study from our lab showed that during mother–adolescent face-to-face interaction, the mother’s frontal region formed multiple inter-brain connections with measured all brain regions of the child: right and left frontal, right and left central, and right and left temporal regions [44]. It was concluded that the mother’s frontal region serves as a “central regulator” of the two-brain dynamics that supports the maturation of the child’s social brain during a period of brain reorganization, such as the transition to adolescence. 

In light of the above, the current study utilized a two-brain perspective to measure the associations between maternal sensitivity and intrusiveness and mother–child inter-brain connectivity patterns. We measured brain-to-brain synchrony during naturalistic face-to-face interactions between mothers and their 5–12-month-old infants, the sensitive period for maturation of the social brain [34,35,36,47]. Interactions were then coded for maternal sensitivity and intrusiveness using a well-validated scheme (coding interactive behavior (CIB) [48]). Three main hypotheses were formulated. First, we expected that mother–infant face-to-face interaction would elicit inter-brain connectivity across widely distributed areas with a special involvement of the mother’s frontal and the infant’s temporal regions. Second, we hypothesized that maternal sensitivity would be associated with greater mother–infant brain-to-brain synchrony, specifically within the mother-frontal–infant-temporal connectivity pattern. Finally, we expected that maternal intrusiveness would be associated with diminished mother–infant frontal–temporal inter-brain synchrony. Overall, our study sought to examine the hypothesis that alterations in online social inputs to the infant’s social brain during its sensitive period of maturation may chart one mechanism by which maternal sensitivity and intrusiveness exert their long-term effect on brain and behavior. 

## 2. Materials and Methods

### 2.1. Participants

All procedures used in this study including paradigms, questionnaires, and equipment were approved by the Reichman University IRB committee and all mothers who signed an informed consent. All procedures were explained to the mothers before the study and were performed in accordance with ethical guidelines. Participants were free to leave the experiment at any time with full compensation. The participants were recruited through online forums and social media groups. Inclusion criteria to participate in the experiment were: the mother is above 18 years of age and received at least 12 years of schooling; mother was not pregnant at the time of the experiment; the infant is the mother’s biological child; both mother and infant are healthy at the time of the experiment; and neither mother nor infant are diagnosed with epilepsy. 

Overall, 150 participants (75 mothers and 75 infants) arrived at the lab. However, only 60 infant–mother dyads had usable dual EEG data from the face-to-face free interaction paradigm (mother; mean age = 33.4 ± 4.0 years, range = 22–42 years, median = 34 years, infants: 25 F, 35 M, mean age = 6.95 ± 1.42 months, range = 4–12 months, median = 6.87 months). All mothers were Caucasian Israeli Jewish. All mothers were of middle-class background and had completed at least high-school education (mean = 16.1 ± 1.8 years of education, median = 16). The birth order of the infants was: 15 infants were first-born, 26 were second-born, and 18 were third-born and up.

### 2.2. Paradigm 

**Figure 1:**
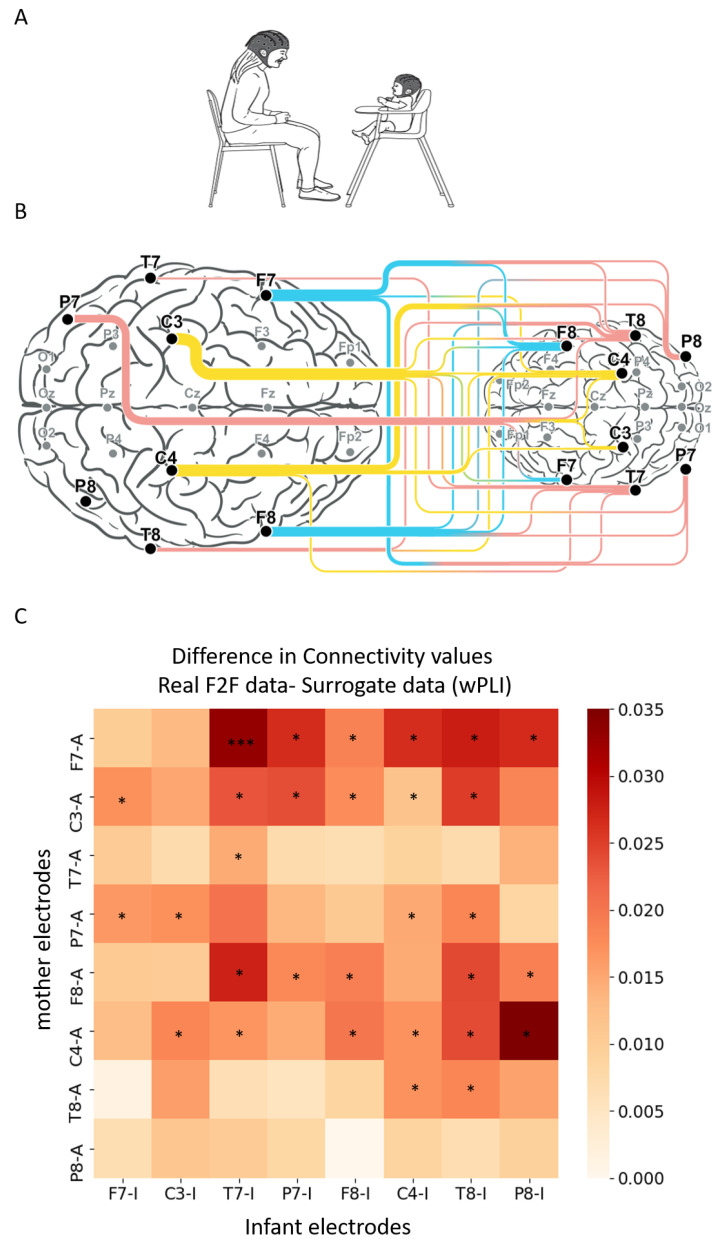
Difference in connectivity values for all electrode combinations between the real face-to-face data and the surrogate data. (**A**) Infant–mother paradigm. Infant and mother were fitted with EEG electrodes and participated in a face-to-face (F2F) free interaction paradigm. (**B**) Illustration of infant–mother inter-brain neural synchrony. Electrodes recorded and analyzed are marked in bold dots; inter-brain neural synchrony values were calculated for theta frequency band (4 to 7 Hz) using weighted phase lag index (wPLI). Connectivity scores were computed for all inter-subject electrode combinations, resulting in 64 wPLI values per dyad. The lines between mother’s (**left**) and infant’s (**right**) electrodes reflect the significant inter-brain connections compared with surrogate data. The width of the lines reflects the number of significant connections each electrode is involved with. (**C**) The x axis represents the infant electrodes, and the y axis the mother electrodes. The color legend represents the difference between the real and the surrogate connectivity scores, such that dark red–colored squares represent comparisons with higher connectivity in the real F2F condition compared with the surrogate data. Nonparametric Mann–Whitney test followed by FDR correction for multiple comparisons revealed real significant neural connectivity during infant–mother face-to-face interaction. The significant comparisons following correction are marked with asterisks.

The mother and the infant entered the experimental room where they were fitted with 17-electrode EEG caps. First, the mother was fitted with the EEG electrodes while the infant was in the same room near the mother. At this point, infants were not required to sit in the experimental chair. The duration of this part was approximately 15 min. Following that part, the infants were fitted with EEG electrodes while they were sitting in a high baby chair or in a baby bouncer chair; alternatively, if needed, they were held by the mothers. The duration of this part was approximately 10 min. Overall, the duration of EEG preparations was ~30 min. During the face-to-face paradigm, infants sat in a high baby chair or in an infant seat (depending on their ability to sit by themselves) (Figure 1A). The mother and the infant were sitting approximately 25 cm from each other. The infant and the mother were left alone in the experiment room and the mother was instructed to play freely with the infant as she typically does. Mothers were instructed to avoid using other objects. Although the mothers were instructed to avoid physical contact with the infants, they were able to touch their infants. We did not instruct the mothers on how to interact with their infants to keep the interaction as natural as possible. We observed that the mothers were singing to the infants, talking with them, playing peak-a-boo, etc. The duration of the interaction was approximately 3 min. All interactions were videotaped for later offline coding.

### 2.3. Dual-EEG Data Acquisition

Neuroelectric activity in both participants of each dyad was simultaneously and continuously recorded using Brain Products GmbH. The system consists of two Acticap helmets with 16 active electrodes arranged according to the international 10/20 system. The reference was fixed on FCz. The impedances were maintained below 10 kΩ. Both subjects were connected to the same amplifier that guaranteed millisecond-range synchrony between the two EEG recordings. 

### 2.4. EEG Preprocessing

Preprocessing was conducted using Python in Anaconda with the MNE software suite. First, for the preprocessing procedure, the EEG data file of each dyad was separated to infant data file and mother data file. Then, a 1–50 HZ band pass filter was applied using forward–backward IIR filter. Next, following segmentation of the signal to 1-s epochs with 500-millisecond overlap between epochs, we applied an automatic algorithm that detect noisy segments. Similar to other hyperscaning EEG research [49], we use the MNE “AutoReject” v0.1 algorithm [50] with Bayesian optimization as the threshold method. Autoreject is an automatic data-driven algorithm for detection and repair of bad segments, using optimal peak-to-peak rejection thresholds subject-wise. Overall, 13.5 ± 16.2% of the epochs were rejected. The percentage of rejected epochs of the mothers was 14.2 ± 18.9% and of the infants was 12.9 ± 13.2%. The autoreject algorithm removes trials containing transient jumps in isolated channels but does not necessarily work well for a systematic physiological artifact that affects multiple sensors. For these purposes, we used MNE’s implementations of FastICA and CORRMAP [51]. CORRMAP allows the manual selection of an independent component (IC) for exclusion in one participant and the use of the chosen component as a template for selecting and excluding similar components in other participants. The general idea behind the CORRMAP algorithm is that artifact patterns are generally similar over a large number of participants. Therefore, correlation between the template IC and each IC solution enables the choosing of the IC with the highest correlation, thus excluding similar components (see Figure S1 for further explanation). Overall, 4.2 ± 1.8 components per subject were detected and removed. For infants, 3.5 ± 1.6 components on average were removed, and for the mothers, 4.8 ± 1.8 components on average were removed.

### 2.5. Connectivity Analysis

Mother–infant inter-brain neural connectivity values were calculated for the theta frequency band, consistent with our previous work of infant–adult inter-brain connectivity [43] focused on the theta frequency band as theta oscillations are implicated in the processing of emotional cues and behavioral states that carry attentional and emotional significance. In infants, moments of social interaction, which involve periods of sustained attention and exploration of novel objects, are associated with increased theta oscillations [52]. Consistent with recent hyperscanning studies [43,44,53], we wished to avoid spurious hyper-connections that could result from similarity in the sensory experiences of the participants and that are not related to the social interaction itself. We therefore decided to use the weighted phase lag index (wPLI) as our measure of inter-brain synchrony, a measure that is an extension of the PLI. By weighing each phase difference according to the magnitude of the lag, phase differences around zero only marginally contribute to the calculation of the wPLI. This procedure reduces the probability of detecting “false positive” connectivity in the case of noise sources with near-zero phase lag and increases the sensitivity in detecting phase synchronization [54]. The wPLI is a robust and widely used method for measuring MEG/EEG functional connectivity. wPLI ranges between 0 and 1, where 0 indicates no synchrony and 1 full synchrony.

Consistent with prior research and to avoid a large number of multiple comparisons, we chose to focus on inter-brain synchrony only in the temporal (T7, T8), occipital-temporal (P7, P8), central (C3, C4), and frontal (F7, F8) regions. These regions have been found in multiple studies [43,44,45,49,55,56,57] to show inter-brain synchrony during ecologically valid social experiments. 

Finally, to eliminate spurious findings and show that the effects stem from “real life” interactions and are not the result of the laboratory settings themselves (i.e., participants experience similar environmental conditions), we compared the neural connectivity values obtained from the real data to those derived from surrogate data generated by randomly pairing mothers and infants from different dyads. For the surrogate data, we created 60 new couples to match the real data. For each electrode pair, the nonparametric Mann–Whitney test was performed between the real dataset and the pair-randomized dataset, followed by FDR correction for multiple comparisons. 

To explore the possibility that artefactual components are contributing to the effects of interest, we conducted a control analysis including the data obtained from the detected (excluded) independent components (“bad” data). We computed wPLI scores using the artefactual components (facial muscles components, eye movement components, and non-physiological components) in the face-to-face paradigm and compared between the real “bad” data to surrogate “bad” data. Same as in the main analysis, connectivity scores were computed for all inter-subject electrode combinations, resulting in 64 wPLI values per dyad. The nonparametric Mann–Whitney test followed by FDR correction for multiple comparisons revealed no significant neural connectivity using the data-detected IC components (all corrected *p* > 0.125) (Figure S2). These results suggest that the reported differences in neural synchrony between the real clean data following removal of all detected components and the surrogate data are not driven by artifacts. 

### 2.6. Maternal Behavioral Profiles; Sensitivity and Intrusiveness

Coding of maternal caregiving profiles was conducted with the Coding Interactive Behavior Manual (CIB; [48]) The CIB is a global rating system of social interaction comprised of multiple scales scored from 1 (low) to 5 (high) that combine into theoretically derived constructs. The CIB has been validated in a large number of studies spanning over 25 countries in healthy and high-risk populations. The system has shown construct validity, test-retest reliability, and prediction to multiple social, cognitive, biological, and neural outcomes [19,58]. The CIB utilizes scales for parent, for infant, and for the dyadic atmosphere.

Sensitivity and intrusiveness are the most widely reported maternal behavioral constellations. The sensitivity and intrusiveness constructs of the CIB have been used in a large number of studies in infancy and showed individual stability from infancy to adolescence [21] and prediction of positive and negative social-emotional outcomes, including mental health, stress, and affiliation hormones [24,26,27,59,60], and brain activation patterns [31,32].

Maternal sensitivity included the following codes: mother acknowledging, mother consistency of style, mother resourcefulness, mother affectionate touch, mother appropriate range of affect, and dyadic adaptation-regulation. Maternal intrusiveness included the following variables: maternal overriding, mother forcing, mother anxiety, and adult-lead interaction. 

Two coders, trained to 85% reliability and blind to all other information, coded the interactions. Interrater reliability, computed for 15% of the interactions, averaged 93% (intraclass *r* = 0.94).

## 3. Results

### 3.1. Inter-Brain Synchrony Increases during Mother–Infant Face-to-Face Interactions

As a first step, we sought to pinpoint inter-brain connectivity patterns associated with mother-infant face-to-face interactions in the theta frequency band (Figure 1A). To demonstrate the existence of neural synchrony during real-life social interactions, we created surrogate data using the methodology described in prior inter-brain research [49,61]. Results revealed 30 significant region-to-region inter-brain connections (out of 64 possible combinations), indicating that inter-brain connectivity is indeed a feature of social interactions and increases during mother–infant face-to-face interaction compared to the surrogate data. These connections involved mainly frontal and central electrodes of the mothers, from both left and right hemispheres (Figure 1B,C). 

Although our analysis was focused on the theta frequency band, to further explore mother–infant neural connectivity patterns, we repeated the same analysis with alpha and beta frequency bands as well. The analysis revealed 11 significant inter-subject connections (17 % of all combinations) for the alpha band and 14 significant inter-subject connections (22% of all combinations) for the beta band (Figure S3). Inter-brain connections in the alpha and beta bands were relatively low in comparison with the number of connections in theta band, and unlike the theta band showed only few connections that involve maternal frontal regions. Still, the analysis revealed that beta and theta frequency bands have several common inter-brain connections, including the mother’s right-central electrode (C4) with the infant’s right-central (C4) and right-temporal (T8, P8) electrodes, as well as connections between the infant’s right-central electrode (C4) and maternal T8, P7, and F7 electrodes. Alpha and theta frequency bands also had few overlapping connections involving the maternal right-central region. 

### 3.2. Links between Inter-Brain Synchrony Patterns and Maternal Sensitivity and Intrusiveness 

Following identification of the various inter-brain connections during mother–infant face-to-face interaction, we examined inter-brain connectivity scores association with the mother’s caregiving profiles—sensitivity (mean = 3.8, SD = 0.61, median = 3.9, range = 2.1–4.8) and intrusiveness (mean = 1.6, SD = 0.78, median = 1.5, range = 1–4.5). Histograms of sensitivity and intrusive scores, as well as the correlation plot between the two measurements (*r* = −0.6, *p* < 0.001), can be found in Figure S4. 

To test whether maternal styles are confounded by demographic factors, we conducted a chi-square test of independence. The analysis revealed that there was no significant association between level of intrusiveness and child birth order (X^2^ (2, N = 52) = 2.6, *p* = 0.27), as well as no significant association between level of sensitivity and child birth order (X^2^ (2, N = 52) =2.7, *p* = 0.26). Moreover, there was no significant association between level of sensitivity and maternal age or infant’s age (both, X^2^ (1, N = 53) = 0.018, *p* = 0.89), and there was no significant association between intrusiveness and maternal or infant’s age (both, X^2^ (1, N = 53) = 0.17, *p* = 0.68). 

Identification of the various inter-brain connections during mother–infant face-to-face interaction reveals that all possible combinations between maternal frontal (both right and left hemisphere) and infant’s temporal (both right and left hemisphere) are significant. The connectivity between mother-frontal and infant-temporal regions has been previously shown in hyperscanning studies of parent–child interaction as prevalent and linked to behavioral measures [41,42,44,45], and therefore, we tested Spearman correlation between the mother-frontal–infant-temporal neural synchrony scores with maternal sensitivity and intrusiveness.

We computed an overall measure of mother-frontal–infant-temporal synchrony by averaging the scores of inter-brain connections of the infant’s temporal and occipito-temporal electrodes (T8-I, T7-I, P8-I, P7-I) with the mother’s frontal electrodes (F7 and F8). We then examined the associations with maternal sensitivity and maternal intrusiveness. 

**Figure 2:**
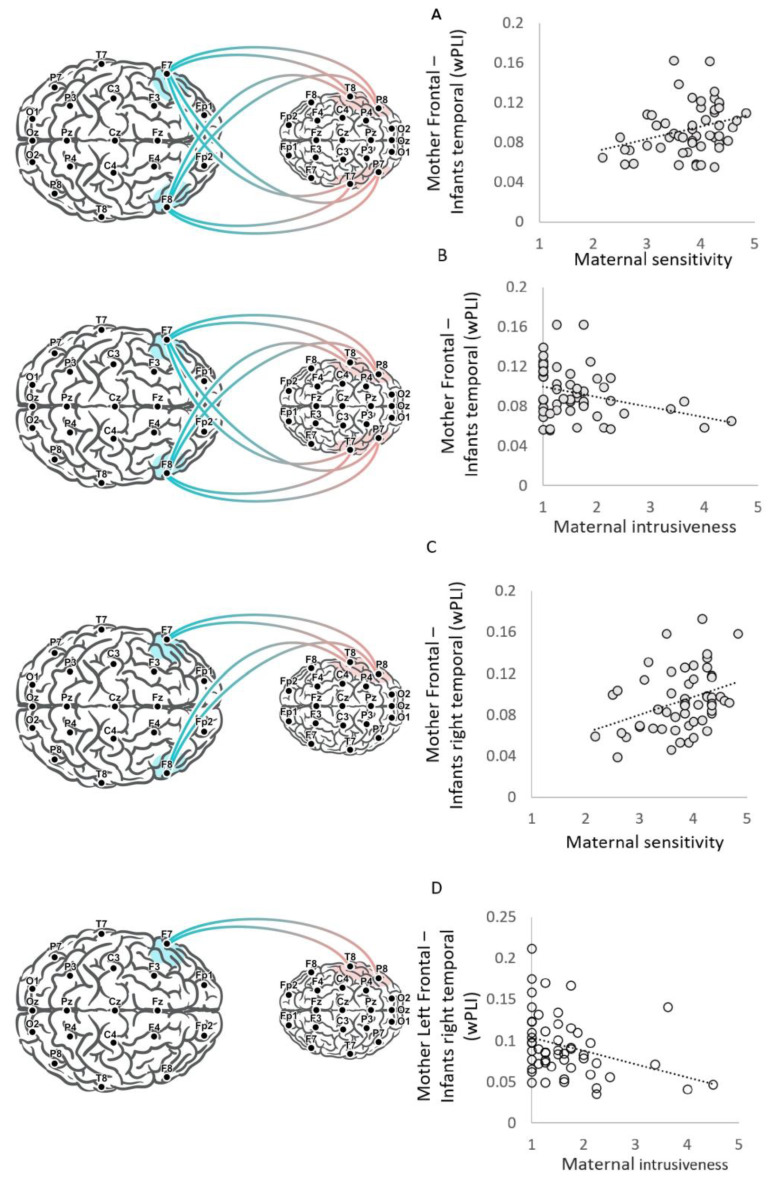
Visualization of brain–behavior correlations. Visualization of the CIB codes of maternal sensitivity and maternal intrusiveness correlations with wPLI values of frontal–temporal inter-brain neural synchrony and frontal–right temporal inter-brain neural synchrony. Mother-frontal–infant-temporal neural synchrony scores correlated with both (**A**) maternal sensitivity scores (N = 53, *r* = 0.31, *p* = 0.023) and (**B**) maternal intrusiveness scores (N = 53, *r* = −0.28, *p* = 0.041). (**C**) Mother-frontal–infant-right-temporal neural synchrony scores correlation with maternal sensitivity scores (N = 53, *r* = 0.34, *p* = 0.013), and (**D**) mother-left-frontal–infant-right-temporal neural synchrony scores correlation with maternal intrusiveness scores (N = 53, *r* = −0.39, *p* = 0.004).

Results revealed that mother-frontal–infant-temporal neural synchrony was positively correlated with maternal sensitivity (N = 53, *r* = 0.31, *p* = 0.023) (Figure 2A) and negatively associated with maternal intrusiveness (N = 53, *r* = −0.28, *p* = 0.041) (Figure 2B).

Since the two maternal styles are significantly inter-correlated, we further explored the obtained correlations accounting the effect of the other maternal style using partial Spearman correlation. The analysis revealed no significant contribution for either sensitivity or intrusiveness in the association with mother-frontal–infant-temporal neural synchrony (sensitivity conditioned on intrusiveness *r* = 0.187, *p* = 0.19; intrusiveness conditioned on sensitivity *r* = −0.125, *p* = 0.38).

This suggest that the more sensitive and less intrusive the mother is, the greater the inter-brain connection between these two areas.

#### 3.2.1. Connectivity with the Infant’s Right and Left Temporal Regions and Brain–Behavior Correlations

To further probe the source of the mother-frontal–infant-temporal connectivity, we conducted post hoc analysis and divided mother–infant connectivity scores to the infant’s right temporal regions and left temporal regions. Results indicate that the mother-frontal–infant-right-temporal synchrony showed positive correlations with maternal sensitivity (N = 53, *r* = 0.34, *p* = 0.013) (Figure 2C) and negative correlations with maternal intrusiveness (N = 53, *r* = −0.37, *p* = 0.006). However, the mother-frontal–infant-left-temporal neural synchrony did not show significant correlations with maternal intrusiveness (*r* = −0.06, *p* = 0.69) or maternal sensitivity (*r* = 0.15, *p* = 0.29). The Fisher Z test between correlations with the infant’s right and left temporal regions revealed a significant difference between the correlations of maternal intrusiveness with infants’ right and left temporal regions (z = −2.1, *p* = 0.019); however, the difference between the brain–behavior linkage with the infant’s right and left temporal regions with maternal sensitivity was not significant (z =1.2, *p* = 0.11). However, when accounting for the other maternal style using a partial Pearson correlation, both maternal styles were not significantly correlated with mother-frontal–infant-right-temporal scores or mother-frontal–infant-left-temporal scores (all *r* < 0.23, all *p* > 0.1).

These findings suggest that inter-brain synchrony between maternal frontal and infants’ right temporal regions is related to both relational patterns, which are interrelated.

#### 3.2.2. Mother’s Right and Left Frontal Regions and Brain–Behavior Correlations

To further explore the source of maternal frontal connectivity, we similarly divided the mothers–infants inter-brain connections to mother-right-frontal–infant-right-temporal and mother-left-frontal–infant-right-temporal and mother-right-frontal–infant-left-temporal and mother-left-frontal–infant-left-temporal.

Results indicate that maternal sensitivity correlated with both mother-right-frontal– infant-right-temporal (N = 53, *r* = 0.27, *p* = 0.048) and mother-left-frontal–infant-right-temporal scores (N = 53, *r* = 0.29, *p* = 0.036). A Fisher Z test between correlations revealed no significant difference between those involving mothers’ left and right frontal connectivity scores (z = −0.1, *p* = 0.46). However, a partial Spearman correlation revealed no significant contribution for sensitivity beyond the intrusiveness in the association with both mother-right-frontal–infant-right-temporal and mother-left-frontal–infant-right-temporal scores (sensitivity conditioned on intrusiveness, both *r* < 0.2, *p* > 0.17).

Maternal intrusiveness scores were negatively correlated with mother-left-frontal–infant-right-temporal scores (*r* = −0.39, *p* = 0.004) (Figure 2D), but not with mother-right- frontal–infant-right-temporal scores (*r* = −0.2, *p* = 0.14). However, a Fisher Z test between correlations revealed no significant difference between the correlations involved mothers’ left and these involve mothers’ right-frontal connectivity scores (z = −1.15, *p* = 0.13). Moreover, a partial Spearman correlation between intrusiveness and mother-left-frontal–infant-right-temporal scores, conditioned on sensitivity, revealed a significant contribution for intrusiveness (*r* = −0.28, *p* = 0.047).

Both maternal intrusiveness and maternal sensitivity were not correlated with mother-right-frontal–infant-left-temporal (sensitivity, *r* = 0.24, *p* = 0.09, intrusiveness *r* = −0.025, *p* = 0.86) or mother-left-frontal–infant-left-temporal (sensitivity, *r* = −0.04, *p* = 0.78, Intrusiveness *r* = −0.03, *p* = 0.83). However, a partial Spearman correlation revealed a significant contribution for sensitivity in the association with mother-right-frontal–infant-left-temporal (*r* = 0.279, *p* = 0.044).

Finally, although the analysis was hypothesis-driven to test association with frontal- temporal regions, Table S1 presents the raw correlation values between all significant electrode connections and maternal styles. Supporting our hypothesis, the only significant correlations involve the inter-brain connections F7-T8, F7-P8 (maternal-left-frontal–infant-right-temporal and occipitotemporal), and F8-P8 (maternal-right-frontal–infants-right-occipitotemporal).

Overall, our findings implicate connectivity between the mother’s frontal regions—both right and left—with the infant’s temporal regions in relation to the mother’s stable behavioral orientation, sensitivity and intrusiveness. We found a specific contribution of intrusiveness to mother-left-frontal–infant-right-temporal synchrony, and a particular contribution of sensitivity to mother-right-frontal–infant-left-temporal synchrony. 

## 4. Discussion

Biobehavioral synchrony, the coordination of physiological processes and behavioral signals between mother and infant during moments of social contact, tunes the infant’s brain to the social world and supports the development of social competencies [62]. Probing this mechanism from a two-brain perspective, we examined the links between patterns of mother–infant inter-brain synchrony during face-to-face interactions using dual-EEG recordings and the two well-studied maternal behavioral orientations—sensitivity and intrusiveness. These behavioral styles have been repeatedly shown to be individually stable across lengthy periods of development and to shape children’s positive (sensitivity) and negative (intrusiveness) socio-emotional outcomes. Our data highlight several important and novel findings. 

First, we found that mother–infant face-to-face communication that builds on the species-typical social cues, including social gaze, vocalizations, positive facial expressions, and natural olfactory cues, elicit greater inter-brain theta synchrony as compared to surrogate data. We found that almost 50% of the possible connectivity combinations (30 out of 64 combinations) were significantly higher during the real mother–infant face-to-face interaction compared to the surrogate data. Prior studies have shown that parental social cues such as gaze [57], maternal vocal and facial expression [61], and olfactory cues [43] have an impact on the degree of neural synchrony. These ostensive social signals are hypothesized to act as synchronizing cues that trigger a transient increase in interpersonal entrainment through phase-resetting processes [57,63,64].

Mother–infant face-to-face interaction elicited inter-brain connections across widely distributed areas, with a particular involvement of the mother’s frontal and central areas and the infant’s temporal regions. These results are in line with previous hyperscanning studies that demonstrated the involvement of temporal and frontal regions in processes of mother–child inter-brain synchrony in research spanning infancy to adolescence [41,42,44,45]. For instance, in an ecological hyperscanning study of adult–infant interactions, we found connectivity with the infant’s right temporal region during interactions with both mother and an unfamiliar female [43]. A dual-MEG setup that employed a social turn-taking verbal imitation task and a passive listening task between mothers and infants showed increased inter-brain synchrony in cortical hubs in the right parietal and bilateral frontal areas. Consistent with our findings, significant inter-brain connections emerged in the theta frequency band between the mother’s frontal regions and the child’s temporal regions [45]. A dual-fNIRS study revealed higher inter-brain synchrony in the bilateral prefrontal and temporo-parietal regions during mother–child cooperation compared to a solitary condition [42]. Similarly, mothers and their 4–6-year-old children exhibited neural synchrony in temporo-parietal and prefrontal areas during a free verbal conversation [41]. During mother–adolescent face-to-face interaction, the mother’s frontal region formed multiple inter-brain connections that were widely distributed across the child’s brain: with the child’s right and left frontal, right and left central, and right and left temporal regions [44]. Consistent with these findings, neural synchrony measured by MEG between mothers and preadolescents (range 9–13 years) was detected in the right superior temporal sulcus (STS) during the observation of own, but not unfamiliar interaction [65]. 

The fronto-temporal network underpins key socio-cognitive functions implicated in social cognition and empathy, including the medial-prefrontal-cortex (mPFC), superior temporal sulcus (STS), superiortemporal-gyrus (STG), insula, inferior-parietal lobule (IPL), and inferior frontal gyrus (IFG) [66,67]. Various brain-imaging studies in which volunteers were asked to perform simple tasks that require taking into account others’ mental states pinpointed similar brain regions, mainly mPFC and the temporo-parietal junction (TPJ), as more active when volunteers make inferences about mental states than compared to inferences about physical or behavioral states [66]. It was further shown that atypical connectivity processes are observed in the fronto-temporal areas in children with autism spectrum disorder and that such disruptions may contribute to the social cognitive deficits of autism [68].

The frontoparietal network undergoes substantial maturation during the first year of life. A recent EEG study in 854 infants assessed at 5 and 10 months investigated social brain development during the first year of life and found that the development of theta-modulated networks progressed from a parietal occipital network in early infancy to a frontoparietal network toward the end of the first year of life. This reconfiguration coincides with the development of selectivity for social versus nonsocial stimuli, with infants approaching the end of their first year showing increased synchronicity of theta communication when observing social videos versus nonsocial videos. This finding suggests the involvement of a frontoparietal theta network in the development of the social brain [36]. Consistent with this frontoparietal theta network maturation pattern, another study examined structural maturation from 3 to 12 months and showed that maturation begins in the primary sensorimotor cortex between 3 and 6 months and continues with the posterior-temporal and frontal regions between 7 and 12 months [34]. This maturational pattern, from sensorimotor to temporal and prefrontal regions, is consistent with other studies that utilized different imaging methods [33,35]. The later maturation of the posterior-temporal region suggests that human-specific social experiences during this sensitive period, such as face-to-face play and imitation games observed universally, may define critical environmental inputs for maturation of this brain region. 

The dense cross-brain linkage deriving from the mother’s frontal cortex is consistent with the well-known mechanism of “external regulation” [69] of the infant’s immature brain by the mother’s mature brain. It has been suggested that the mother’s external regulation of the infant’s developing neurobiological systems during early sensitive periods represents the critical factor that shapes the experience-dependent growth of brain areas [70] and tunes the child’s immature brain to social life through inter-brain mechanisms embedded within coordinated social behavior [71]. We suggest that the neural linkage between the mother’s frontal and the infant’s temporal region, as embedded within real-life social moments, contributes to maturation of the infant’s social frontoparietal theta network during the first year of life when this network undergoes rapid development. 

Mother–infant inter-brain neural connectivity values were calculated for the theta frequency band, consistent with our previous work on infant–adult inter-brain connectivity [43]. Theta oscillations are implicated in the processing of emotional cues and behavioral states that carry attentional and emotional significance [52]. However, to further explore mother–infant neural connectivity patterns we repeated the same analysis with a focus on alpha and beta frequency bands. It was found that the number of inter-brain connections in the alpha and beta bands was relatively low in comparison with the number of connections in the theta band, and these results are supported by previous studies showing the involvement of multiple oscillatory rhythms across distributed areas in the processing of attachment-related cues [29]. Notably, inter-brain connections that integrated both beta and theta, as well as both alpha and theta, were common primarily in connections involving the mother’s right central region. The involvement of the mother’s right central region in mother–infant brain-to-brain synchrony during naturalistic interaction accords with our previous findings of infant–adult inter-brain connectivity [43]. 

The second important aspect of the current study highlights the brain–behavior link as investigated from a two-brain perspective. Results show that higher maternal sensitivity and lower intrusiveness are associated with higher mother–infant brain-to-brain synchrony, and that the strongest correlation involves the mother-frontal–infant-right-temporal connectivity scores. Maternal sensitivity to the infant’s social cues during direct communication enables these precious moments of mother–infant coordination to frame the connectivity between their two brains. Our findings are the first to show that moments of sensitive caregiving are reflected in inter-brain synchrony between the mother’s frontal regions and the child’s temporal regions. Studies have shown that the effects of maternal sensitivity extend into adulthood and sensitivity experienced during the first three years of life carries persistent associations with social and academic competence through age 32 years [6]. Our findings chart inter-brain synchrony as one mechanism by which maternal sensitivity may exert such long-term, broad-band effect. 

Sensitivity and intrusiveness are two distinct profiles of maternal caregiving that are negatively correlated to each other. Sensitivity is composed of parameters related directly to “not being intrusive”, such as a mother acknowledging the infant’s signals, but also includes “positive” components of the species-typical maternal social behavior, such as gaze, affect, and affectionate touch. This may have accounted for the findings that these two styles were not uniquely related to the mother-frontal–infant-right-temporal connectivity scores. Accounting for the contribution of intrusiveness, we found that maternal sensitivity had a moderate yet significant positive association with the mother-right-frontal–infant-left-temporal connectivity scores. This may suggest that this inter-brain link is specific to the “positive” components of the sensitivity construct and not to the “non-intrusive” components. Interestingly, a recent mother–child hyperscanning study showed that the extent of child engagement and empathy toward the mother affected this link between the mother’s right frontal area and the child’s left temporal area [44], highlighting this connection as particularly sensitive to the positive, engaged, and empathic elements in the dyadic relationship.

In contrast to the findings for sensitivity, higher maternal intrusiveness was associated with diminished mother–infant inter-brain synchrony, where the strongest effect was found in the mother-left-frontal–infant-right-temporal connectivity, even after when accounting for the contribution of sensitivity. Maternal intrusiveness consists of various aspects related to maternal control: over-stimulation, anxiety, and disregard of the infant’s pace and rhythms. Our findings are consistent with a previous hyperscanning study showing that high levels of maternal self-reported stress were negatively associated with the degree of mother–child neural synchrony during a cooperation interaction [42]. Mothers with anxiety disorders typically express adequate amounts of positive interpersonal elements, but these are not adapted to the infant’s cues [72]. Anxious mothers may even display more social behavior than controls on some interactive components, such as ‘motherese’ vocalizations and the display of positive affect. However, the mother’s behavior is typically not matched to the infant’s state and signals, and mothers often maintain high-pitched, sing-song vocalizations for much of the interaction, regardless of whether the infant is socially responsive, gaze averting, or showing signs of fatigue [72,73]. We found that maternal intrusiveness was more strongly correlated with the mother-left-frontal–infant-right-temporal inter-brain synchrony rather than connectivity scores involving the right frontal area. The frontal electrodes (F7, F8) record activity from both the anterior temporal and frontal regions, specifically the inferior frontal gyrus (IFG). The left IFG contains Broca’s area, which is a key region for language comprehension and production. Inter-brain synchrony that involves a key region for language comprehension may have a vital role in language acquisition during early development. 

The right temporal and occipitotemporal cortex comprises several brain regions, including the fusiform gyrus [41] and the superior temporal sulcus (STS), and serves as a key node of the social brain implicated in simulation, mentalization, and action observation [42]. The STS is involved in different aspects of social cognition and language processing and shows the greatest response to meaningful stimuli of communicative significance. A species-specific voice-selective region has been identified in the right STS that responds more strongly to human vocal sounds compared to a variety of non-vocal sounds. However, the STS also activates in response to a wide range of non-verbal signals used in communication, such as eye gaze and biological motion. These findings raise the possibility that the voice-selective region of the STS may be especially sensitive to vocal sounds that are communicative, rather than to all human vocal sounds [74]. It has been suggested that the right STS is particularly critical in early language learning because the functions of the right STS are the communicative skills available to the pre- or peri-linguistic child [75], and studies highlight the importance of early mother–child interaction for children’s language development [76]. Intrusiveness is mostly observed during parent–infant play and refers to the parent’s tendency to control the situation and to interfere with or override the infant’s activities rather than follow the infant’s “lead” [77], thereby reducing the child’s verbal response opportunities and disrupting language learning, and maternal intrusiveness has indeed been linked with disruptions to language development [16,17,18]. Our findings suggest that a possible mechanism for disrupted language development associated with maternal intrusiveness is the reduced left-frontal–right-temporal inter-brain connection, which taps regions involved in language production and perception. This is supported by an infant–mother dual-MEG study that highlights the important role of turn-taking during mother–infant verbal interactions and suggests a role for social “gating” in language learning [45]. 

Several study limitations should be acknowledged. First, while exact localization of neural regions is not possible with EEG, and future research using other methods such as fNIRS or dual-MEG is required to pinpoint the brain areas detected here with greater specificity, our results highlight connectivity between infants’ temporal and mothers’ frontal regions, which are also found to be associated with maternal profiles measurements. Second, in the current study, we compared mother–infant face-to-face interaction to surrogate data. This analysis does not control for the genetic similarity in brain patterns that might emerge between a mother and her child irrespective of the interaction. To tease apart the biological component that may contribute to future inter-brain synchrony research, one should compare the synchrony among women who had a child by donor egg in vitro fertilization with mother–infant dyads that went through IVF but with their own egg. Moreover, although the current study focused on mother–infant interactions, we do not hypothesize that the effects are specific to biological mothers and future studies with fathers, as well as male and female non-parent caregivers (e.g., grandparents, childcare providers) and strangers are needed for the generalization of the findings. Finally, it is important to remember that our results are correlational and we in no way infer causality. These findings raise the possibility that more sensitive interactions exert long-term effects on child brain and behavior via inter-brain mechanisms, but this hypothesis should be validated in longitudinal and intervention studies. 

## 5. Conclusions

In summary, the neural mechanisms by which maternal sensitivity and intrusiveness exert their lasting impact on the child’s brain and behavior are not fully clear and ours is the first study to examine the links between these well-researched maternal styles and inter-brain synchrony during naturalistic interactions. Our results highlight the involvement of the mother’s frontal regions, both right and left, and the infant’s right and left temporal regions in mother–infant inter-brain synchrony during direct communication and show that higher maternal sensitivity is associated with greater mother–infant inter-brain synchrony, while higher maternal intrusiveness links with lower neural coordination. Further two-brain research is needed to examine how well-adapted and sensitive caregiving creates a long-term and measurable effect on the infant’s neurodevelopment, cognitive growth, social-emotional competencies, language development, and maturation of the social brain.

## Data Availability

The data that support the findings of this study are available on request from the corresponding authors. The data are not publicly available because of their nature—videos of interactions containing information that could compromise the privacy of research participants.

## Supplementary Materials

The following supporting information can be downloaded at https://www.mdpi.com/article/10.3390/biology12020284/s1, Figure S1: ICA Detection and exclusion procedure, Figure S2: Exploring the possibility that artefactual components are contributing to the effects of interest, Figure S3: Delta Connectivity during mother-infant free-interaction in alpha and beta frequency bands, Figure S4: Maternal Intrusiveness and Maternal Sensitivity distribution, Table S1: Maternal styles correlations with all significant electrode combinations.

## References

[1] Ainsworth M.D.S., Blehar M.C., Waters E., Wall S., Hillsdale N.J. (1978). Patterns of Attachment: A Psychological Study of the Strange Situation.

[2] van Ijzendoorn M., Kroonenberg P.M. (1988). Cross-Cultural Patterns of Attachment: A Meta-Analysis of the Strange Situation. Child Dev..

[3] De Wolff M.S., van Ijzendoorn M.H. (1997). Sensitivity and Attachment: A Meta-Analysis on Parental Antecedents of Infant Attachment. Child Dev..

[4] Bakermans-Kranenburg M.J., van Ijzendoorn M.H., Juffer F. (2003). Less is More: Meta-Analyses of Sensitivity and Attachment Interventions in Early Childhood. Psychol. Bull..

[5] Stams G.-J.J.M., Juffer F., van Ijzendoorn M.H. (2002). Maternal sensitivity, infant attachment, and temperament in early childhood predict adjustment in middle childhood: The case of adopted children and their biologically unrelated parents. Dev. Psychol..

[6] Raby K.L., Roisman G.I., Fraley R.C., Simpson J.A. (2015). The Enduring Predictive Significance of Early Maternal Sensitivity: Social and Academic Competence Through Age 32 Years. Child Dev..

[7] Leerkes E.M., Blankson A.N., O’Brien M. (2009). Differential Effects of Maternal Sensitivity to Infant Distress and Nondistress on Social-Emotional Functioning. Child Dev..

[8] Feldman R., Eidelman A.I., Rotenberg N. (2004). Parenting stress, infant emotion regulation, maternal sensitivity, and the cognitive development of triplets: A model for parent and child influences in a unique ecology. Child Dev..

[9] Fraley R.C., Roisman G.I., Haltigan J.D. (2013). The legacy of early experiences in development: Formalizing alternative models of how early experiences are carried forward over time. Dev. Psychol..

[10] Olson S.L., Bates J.E., Sandy J.M., Lanthier R. (2000). Early developmental precursors of externalizing behavior in middle childhood and adolescence. J. Abnorm. Child Psychol..

[11] Shaw D.S., Keenan K., Vondra J.I. (1994). Developmental Precursors of Externalizing Behavior: Ages 1 to 3. Dev. Psychol..

[12] Egeland B., Farber E.A. (1984). Infant-mother attachment: Factors related to its development and changes over time. Child Dev..

[13] Park K.A., Waters E. (1989). Security of attachment and preschool friendships. Child Dev..

[14] Isabella R.A., Belsky J. (1991). Interactional Synchrony and the Origins of Infant-Mother Attachment: A Replication Study. Child Dev..

[15] Erickson M.F., Sroufe L.A., Egeland B. (1985). The Relationship between Quality of Attachment and Behavior Problems in Preschool in a High-Risk Sample. Monogr. Soc. Res. Child Dev..

[16] Nelson K. (1973). Structure and Strategy in Learning to Talk. Monogr. Soc. Res. Child Dev..

[17] Keown L.J., Woodward L.J., Field J. (2001). Language Development of Pre-School Children Born to Teenage Mothers. Infant Child Dev..

[18] Conway L.J., Levickis P., Smith J., Mensah F., Wake M., Reilly S. (2018). Maternal communicative behaviours and interaction quality as predictors of language development: Findings from a community-based study of slow-to-talk toddlers. Int. J. Lang. Commun. Disord..

[19] Feldman R. (2021). Social Behavior as a Transdiagnostic Marker of Resilience. Annu. Rev. Clin. Psychol..

[20] Belsky J., Taylor D.G. (1984). The Pennsylvania Infant and Family Development Project, III: The origins of individual differences in infant-mother attachment: Maternal and infant contributions. Child Dev..

[21] Feldman R. (2010). The relational basis of adolescent adjustment: Trajectories of mother–child interactive behaviors from infancy to adolescence shape adolescents’ adaptation. Attach. Hum. Dev..

[22] Yaniv A.U., Salomon R., Waidergoren S., Shimon-Raz O., Djalovski A., Feldman R. (2021). Synchronous caregiving from birth to adulthood tunes humans’ social brain. Proc. Natl. Acad. Sci. USA.

[23] Ulmer-Yaniv A., Djalovski A., Yirmiya K., Halevi G., Zagoory-Sharon O., Feldman R. (2018). Maternal immune and affiliative biomarkers and sensitive parenting mediate the effects of chronic early trauma on child anxiety. Psychol. Med..

[24] Yirmiya K., Motsan S., Zagoory-Sharon O., Feldman R. (2020). Human attachment triggers different social buffering mechanisms under high and low early life stress rearing. Int. J. Psychophysiol..

[25] Feldman R., Gordon I., Zagoory-Sharon O. (2011). Maternal and paternal plasma, salivary, and urinary oxytocin and parent-infant synchrony: Considering stress and affiliation components of human bonding. Dev. Sci..

[26] Yirmiya K., Djalovski A., Motsan S., Zagoory-Sharon O., Feldman R. (2018). Stress and immune biomarkers interact with parenting behavior to shape anxiety symptoms in trauma-exposed youth. Psychoneuroendocrinology.

[27] Ulmer-Yaniv A., Djalovski A., Priel A., Zagoory-Sharon O., Feldman R. (2018). Maternal depression alters stress and immune biomarkers in mother and child. Depress. Anxiety.

[28] Pratt M., Goldstein A., Levy J., Feldman R. (2017). Maternal Depression Across the First Years of Life Impacts the Neural Basis of Empathy in Preadolescence. J. Am. Acad. Child Adolesc. Psychiatry.

[29] Pratt M., Goldstein A., Feldman R. (2018). Child brain exhibits a multi-rhythmic response to attachment cues. Soc. Cogn. Affect. Neurosci..

[30] Levy J., Goldstein A., Feldman R. (2019). The neural development of empathy is sensitive to caregiving and early trauma. Nat. Commun..

[31] Zeev-Wolf M., Dor-Ziderman Y., Pratt M., Goldstein A., Feldman R. (2022). Investigating default mode network connectivity disruption in children of mothers with depression. Br. J. Psychiatry.

[32] Zeev-Wolf M., Levy J., Goldstein A., Zagoory-Sharon O., Feldman R. (2019). Chronic Early Stress Impairs Default Mode Network Connectivity in Preadolescents and Their Mothers. Biol. Psychiatry Cogn. Neurosci. Neuroimaging.

[33] Deoni S.C.L., Mercure E., Blasi A., Gasston D., Thomson A., Johnson M., Williams S.C.R., Murphy D.G.M. (2011). Mapping infant brain myelination with magnetic resonance imaging. J. Neurosci..

[34] Lemaître H., Augé P., Saitovitch A., Vinçon-Leite A., Tacchella J.-M., Fillon L., Calmon R., Dangouloff-Ros V., Lévy R., Grévent D. (2020). Rest Functional Brain Maturation during the First Year of Life. Cereb. Cortex.

[35] Gao W., Alcauter S., Elton A., Hernandez-Castillo C.R., Smith J.K., Ramirez J., Lin W. (2015). Functional network development during the first year: Relative sequence and socioeconomic correlations. Cereb. Cortex.

[36] van der Velde B., White T., Kemner C. (2021). The emergence of a theta social brain network during infancy. Neuroimage.

[37] Feldman R. (2012). Parent-infant synchrony: A biobehavioral model of mutual influences in the formation of affiliative bonds. Monogr. Soc. Res. Child Dev..

[38] Feldman R. (2016). The neurobiology of mammalian parenting and the biosocial context of human caregiving. Horm. Behav..

[39] Feldman R. (2020). What is resilience: An affiliative neuroscience approach. World Psychiatry.

[40] Feldman R. (2017). The Neurobiology of Human Attachments. Trends Cogn. Sci..

[41] Nguyen T., Schleihauf H., Kayhan E., Matthes D., Vrtička P., Hoehl S. (2021). Neural synchrony in mother–child conversation: Exploring the role of conversation patterns. Soc. Cogn. Affect. Neurosci..

[42] Nguyen T., Schleihauf H., Kayhan E., Matthes D., Vrtička P., Hoehl S. (2019). The effects of interaction quality on neural synchrony during mother-child problem solving. Cortex.

[43] Endevelt-Shapira Y., Djalovski A., Dumas G., Feldman R. (2021). Maternal chemosignals Enhance Infant-Adult Brain-to-Brain Synchrony. Sci. Adv..

[44] Schwartz L., Levy J., Endevelt-Shapira Y., Djalovski A., Hayut O., Dumas G., Feldman R. (2022). Technologically-assisted communication attenuates inter-brain synchrony. Neuroimage.

[45] Lin J.-F.L., Imada T., Meltzoff A.N., Hiraishi H., Ikeda T., Takahashi T., Hasegawa C., Yoshimura Y., Kikuchi M., Hirata M. (2022). Dual-MEG interbrain synchronization during turn-taking verbal interactions between mothers and children. Cereb. Cortex.

[46] Kaitz M., Maytal H. (2005). Interactions between anxious mothers and their infants: An integration of theory and research findings. Infant Ment. Health J..

[47] Feldman R. (2015). Sensitive periods in human social development: New insights from research on oxytocin, synchrony, and high-risk parenting. Dev. Psychopathol..

[48] Feldman R. (1998). Coding Interactive Behavior.

[49] Djalovski A., Dumas G., Kinreich S., Feldman R. (2020). Human Attachments Shape Interbrain Synchrony toward Efficient Performance of Social Goals. Neuroimage.

[50] Jas M., Engemann D.A., Bekhti Y., Raimondo F., Gramfort A. (2017). Autoreject: Automated artifact rejection for MEG and EEG data. Neuroimage.

[51] Viola F.C., Thorne J., Edmonds B., Schneider T.R., Eichele T., Debener S. (2009). Semi-automatic identification of independent components representing EEG artifact. Clin. Neurophysiol..

[52] Orekhova E., Stroganova T., Posikera I., Elam M. (2006). EEG theta rhythm in infants and preschool children. Clin. Neurophysiol..

[53] Dikker S., Michalareas G., Oostrik M., Serafimaki A., Kahraman H.M., Struiksma M.E., Poeppel D. (2021). Crowdsourcing neuroscience: Inter-brain coupling during face-to-face interactions outside the laboratory. Neuroimage.

[54] Vinck M., Oostenveld R., van Wingerden M., Battaglia F., Pennartz C.M.A. (2011). An improved index of phase-synchronization for electrophysiological data in the presence of volume-conduction, noise and sample-size bias. NeuroImage.

[55] Kinreich S., Djalovski A., Kraus L., Louzoun Y., Feldman R. (2017). Brain-to-Brain Synchrony during Naturalistic Social Interactions. Sci. Rep..

[56] Hirsch J., Zhang X., Noah J.A., Ono Y. (2017). Frontal temporal and parietal systems synchronize within and across brains during live eye-to-eye contact. Neuroimage.

[57] Leong V., Byrne E., Clackson K., Georgieva S., Lam S., Wass S. (2017). Speaker gaze increases information coupling between infant and adult brains. Proc. Natl. Acad. Sci. USA.

[58] Feldman R., Mayes L., Lewis M. (2012). Parenting Behavior as the Environment Where Children Grow. The Cambridge Handbook of Environment in Human Development.

[59] Feldman R., Eidelman A.I. (2009). Biological and environmental initial conditions shape the trajectories of cognitive and social-emotional development across the first years of life. Dev. Sci..

[60] Pratt M., Zeev-Wolf M., Goldstein A., Feldman R. (2019). Exposure to early and persistent maternal depression impairs the neural basis of attachment in preadolescence. Prog. Neuro-Psychopharmacol. Biol. Psychiatry.

[61] Santamaria L., Noreika V., Georgieva S., Clackson K., Wass S., Leong V. (2020). Emotional valence modulates the topology of the parent-infant inter-brain network. Neuroimage.

[62] Feldman R. (2012). Bio-behavioral synchrony: A model for integrating biological and microsocial behavioral processes in the study of parenting. Parenting.

[63] Wass S.V., Whitehorn M., Haresign I.M., Phillips E., Leong V. (2020). Interpersonal Neural Entrainment during Early Social Interaction. Trends Cogn. Sci..

[64] Carozza S., Leong V. (2021). The Role of Affectionate Caregiver Touch in Early Neurodevelopment and Parent–Infant Interactional Synchrony. Front. Neurosci..

[65] Levy J., Goldstein A., Feldman R. (2017). Perception of social synchrony induces mother–child gamma coupling in the social brain. Soc. Cogn. Affect. Neurosci..

[66] Frith U., Frith C. (2001). The biological basis of social interaction. Curr. Dir. Psychol. Sci..

[67] Atzil S., Hendler T., Zagoory-Sharon O., Winetraub Y., Feldman R. (2012). Synchrony and specificity in the maternal and the paternal brain: Relations to oxytocin and vasopressin. J. Am. Acad. Child Adolesc. Psychiatry.

[68] Urbain C., Vogan V.M., Ye A.X., Pang E.W., Doesburg S.M., Taylor M.J. (2016). Desynchronization of fronto-temporal networks during working memory processing in autism. Hum. Brain Mapp..

[69] A Hofer M. (1994). Early relationships as regulators of infant physiology and behavior. Acta Paediatr..

[70] Cirulli F., Berry A., Alleva E. (2003). Early disruption of the mother–infant relationship: Effects on brain plasticity and implications for psychopathology. Neurosci. Biobehav. Rev..

[71] Feldman R. (2015). The adaptive human parental brain: Implications for children’s social development. Trends Neurosci..

[72] Feldman R., Granat A., Pariente C., Kanety H., Kuint J., Gilboa-Schechtman E. (2009). Maternal Depression and Anxiety Across the Postpartum Year and Infant Social Engagement, Fear Regulation, and Stress Reactivity. J. Am. Acad. Child Adolesc. Psychiatry.

[73] Feldman R. (2007). Parent-infant synchrony and the construction of shared timing; physiological precursors, developmental outcomes, and risk conditions. J. Child Psychol. Psychiatry.

[74] Shultz S., Vouloumanos A., Pelphrey K. (2012). The Superior Temporal Sulcus Differentiates Communicative and Noncommunicative Auditory Signals. J. Cogn. Neurosci..

[75] Redcay E. (2008). The superior temporal sulcus performs a common function for social and speech perception: Implications for the emergence of autism. Neurosci. Biobehav. Rev..

[76] Kuhl P.K. (2010). Brain Mechanisms in Early Language Acquisition. Neuron.

[77] Huffmeijer R., Bakermans-Kranenburg M.J., Gervain J. (2020). Maternal intrusiveness predicts infants’ event-related potential responses to angry and happy prosody independent of infant frontal asymmetry. Infancy.

