# Supplementary material for: Mother–Infant Brain-to-Brain Synchrony Patterns Reflect Caregiving Profiles
Source: Biology (Basel). 2023 Feb 10;12(2):284. doi: 10.3390/biology12020284 (PMC9953313; doi:10.3390/biology12020284)
Supplement: Supplementary file 1 [file biology-12-00284-s001.zip › biology-2145031-supplementary.pdf]

Supplementary Figures

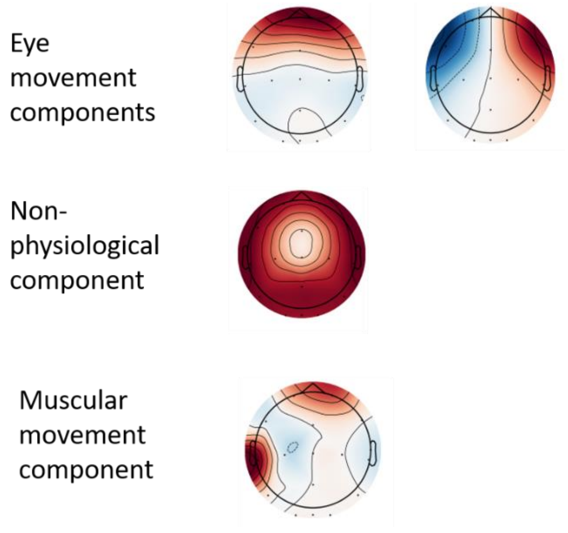

Figure S1.ICA Detection and exclusion procedure

Following the autoreject step**,** we identified and excluded facial muscles components, eye movement components and non – physiological components. The identification was based on topographies and power spectrum. Importantly, the selected templates were balanced across hemispheres. Following the manual selection of templates, we applied the MNE's implementations of FastICA and CORRMAP. CORRMAP allows to manually select an independent component (IC) for one participant and use the chosen component as a template for selecting similar components in other participants.

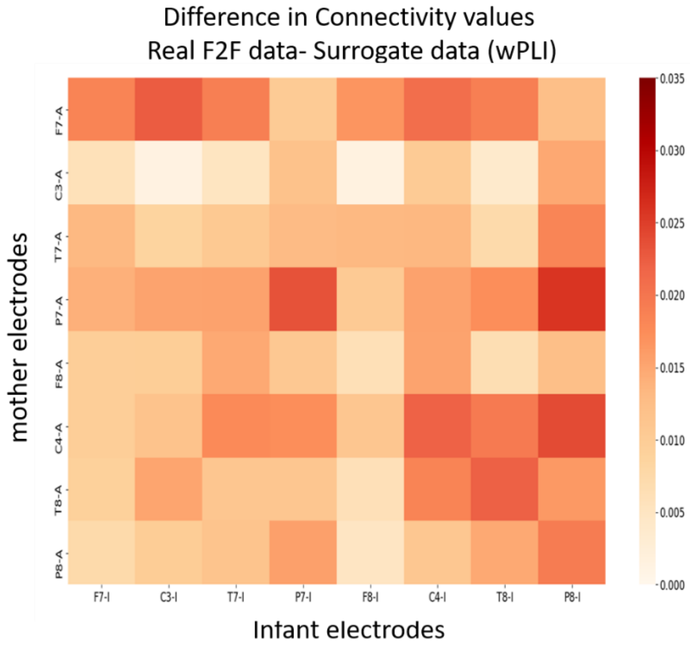

Figure S2. Exploring the possibility that artefactual components are contributing to the effects of interest.

To explore the possibility that artefactual components are contributing to the effects of interest, we included the data obtained from the selected components ("bad" data). We computed wPLI scores using the artefactual components (facial muscles components, eye movement components and non – physiological components) in the face-to-face paradigm and compared between the real “bad” data to surrogate “bad” data. Same as the main analysis, Connectivity scores were computed for all inter-subject electrode combinations, resulting in 64 wPLI values per dyad. The x axis represents the infant electrodes, and the y axis the mother electrodes. Nonparametric Mann-Whitney test followed by FDR correction for multiple comparisons revealed no significant neural connectivity using the “bad” data (all corrected $p > 0.125$). These results suggest that the reported differences in neural synchrony between the real clean data following removal of all detected components and the surrogate data are not driven by artifacts

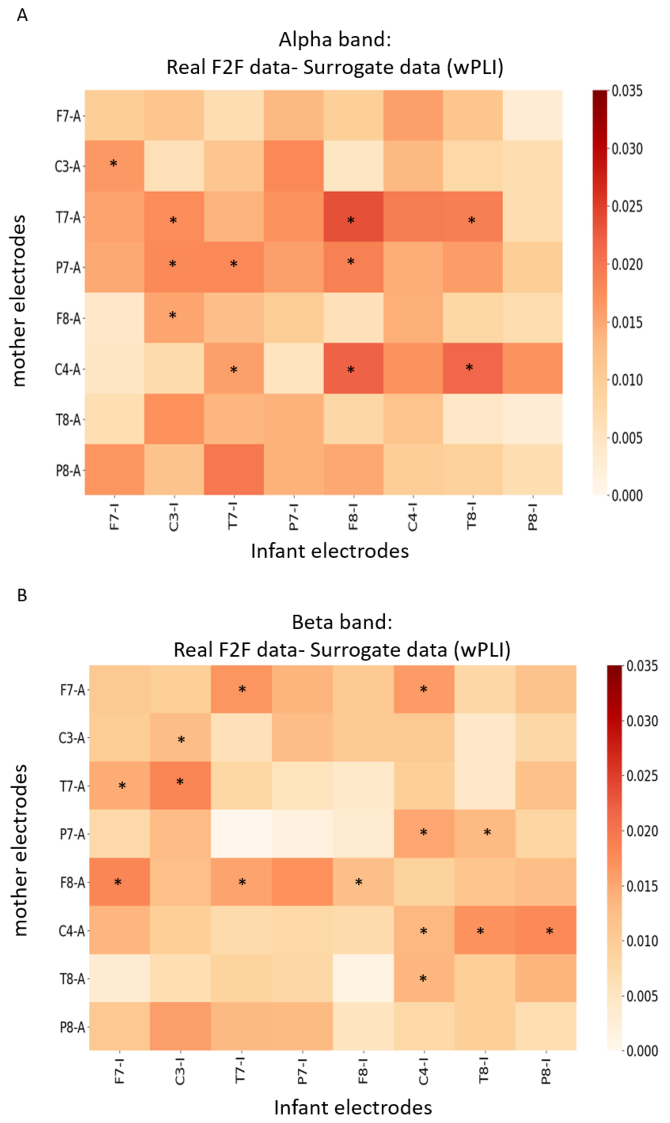

Figure S3. Delta Connectivity during mother-infant free-interaction in alpha and beta frequency bands.

Difference in connectivity values for all electrode combinations between the real face-to-face data and the surrogate data in (A) alpha (8-12 Hz) and (B) beta (13-20 Hz) frequency bands. inter-brain neural synchrony values were calculated for alpha (8-12 Hz) and (B) beta (13-20 Hz) frequency bands using weighted phase lag index (wPLI). Connectivity scores were computed for all inter-subject electrode combinations, resulting in 64 wPLI values per dyad. The x axis represents the infant electrodes, and the y axis the mother electrodes. Nonparametric Mann-Whitney test followed by FDR correction for multiple comparisons revealed 11 significant inter-subject connections (17 % of all combinations) in the alpha band and 14 connections (22 % of all combinations) in the beta band. The significant comparisons following correction are marked with asterisks.

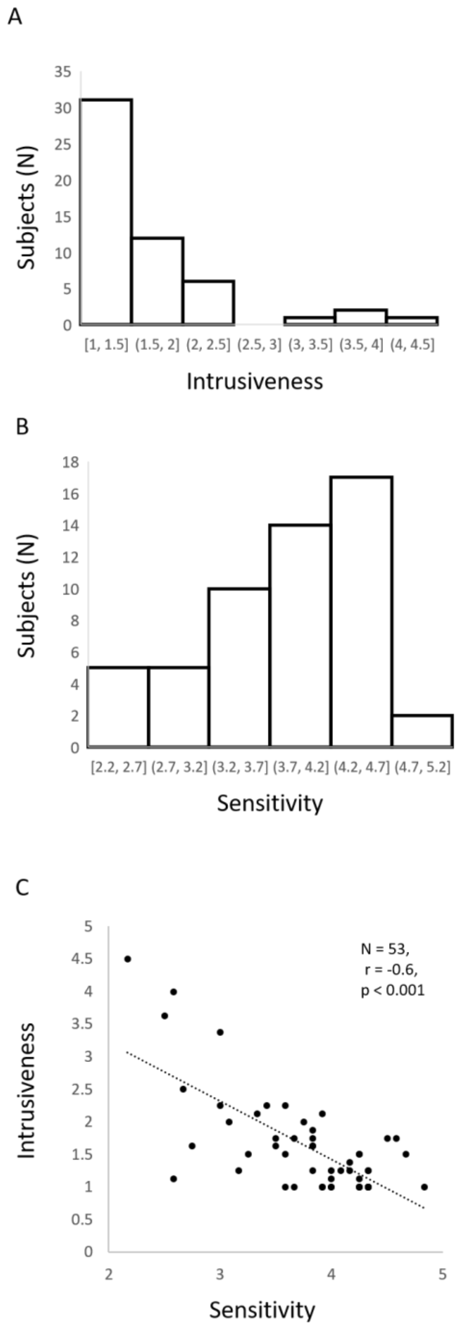

Figure S4. Maternal Intrusiveness and Maternal Sensitivity distribution.

Distribution of (A)Maternal Intrusiveness and (B) maternal Sensitivity scores(C)and Pearson correlation between maternal sensitivity and maternal intrusiveness scores.

Table S1. Maternal styles correlations with all significant electrode combinations

	Spearman's rho sensitivity	P sensitivity	Spearman's rho intrusiveness	P intrusiveness
F7-1T7-0	-0.028	0.843	-0.066	0.641
F7-1P7-0	-0.016	0.908	-0.024	0.864
F7-1F8-0	0.065	0.644	-0.186	0.183
F7-1C4-0	-0.095	0.5	-0.006	0.967
**F7-1T8-0***	0.192	0.169	**-0.356**	**0.009***
**F7-1P8-0***	**0.258**	**0.062**	**-0.308**	**0.025***
C3-1F7-0	-0.017	0.901	-0.142	0.311
C3-1T7-0	-0.046	0.744	-0.047	0.739
C3-1P7-0	0.015	0.916	-0.172	0.217
C3-1F8-0	-0.089	0.525	-0.084	0.549
C3-1C4-0	0.037	0.791	-0.065	0.644
C3-1T8-0	0.045	0.751	-0.263	0.057
T7-1T7-0	0.011	0.938	0.211	0.13
P7-1F7-0	0.008	0.954	0.004	0.977
P7-1C3-0	0.065	0.645	0.235	0.091
P7-1C4-0	-0.039	0.782	0.047	0.736
P7-1T8-0	0.25	0.072	-0.082	0.557
F8-1T7-0	0.145	0.299	-0.003	0.983
F8-1P7-0	0.181	0.194	-0.025	0.858
F8-1F8-0	0.108	0.443	-0.004	0.975
F8-1T8-0	0.165	0.237	-0.048	0.735
**F8-1P8-0***	**0.34**	**0.013***	**-0.325**	**0.017***
C4-1C3-0	0.061	0.666	0.204	0.143
C4-1T7-0	0.048	0.735	0.158	0.259
C4-1F8-0	0.137	0.326	0.056	0.693
C4-1C4-0	-0.146	0.295	0.061	0.664
C4-1T8-0	0.24	0.083	0.026	0.852
C4-1P8-0	0.18	0.197	-0.049	0.728
T8-1C4-0	-0.016	0.911	0.008	0.954
T8-1T8-0	0.07	0.618	-0.038	0.787

* $p < 0.05$
